
JOURNAL INFORMATION
==============================
NLM Title Abbreviation: Biospektrum (Heidelb)
Journal ID: Biospektrum (Heidelb)
NLM Journal ID: 9615685

Biospektrum

ISSN: 0947-0867
EISSN: 1868-6249

ARTICLE INFORMATION
==============================
PMCID: PMC7985589
PMID: 33776218
DOI: 10.1007/s12268-021-1554-z
Article ID: 1554
Article version: 1
Subjects: Wissenschaft · Special: High Content Imaging

Neue Parameter für die Wirkstofftestung gegen Trypanosoma cruzi

Fesser Anna
Kaiser Marcel
Mäser Pascal pascal.maeser@swisstph.ch

grid.6612.3 0000 0004 1937 0642 Universität Basel Schweizerisches Tropen- und Public Health-Institut (Swiss TPH), Socinstraße 57, CH-4051 Basel, Schweiz
Electronic publication date: 2021 Mar 23
Print publication date: 2021
Volume: 27
Issue: 2
First page: 168
Last page: 170
Copyright: © Die Autoren 2021
License: Open Access: Dieser Artikel wird unter der Creative Commons Namensnennung 4.0 International Lizenz veröffentlicht, welche die Nutzung, Vervielfältigung, Bearbeitung, Verbreitung und Wiedergabe in jeglichem Medium und Format erlaubt, sofern Sie den/die ursprünglichen Autor(en) und die Quelle ordnungsgemäß nennen, einen Link zur Creative Commons Lizenz beifügen und angeben, ob Änderungen vorgenommen wurden. Die in diesem Artikel enthaltenen Bilder und sonstiges Drittmaterial unterliegen ebenfalls der genannten Creative Commons Lizenz, sofern sich aus der Abbildungslegende nichts anderes ergibt. Sofern das betreffende Material nicht unter der genannten Creative Commons Lizenz steht und die betreffende Handlung nicht nach gesetzlichen Vorschriften erlaubt ist, ist für die oben aufgeführten Weiterverwendungen des Materials die Einwilligung des jeweiligen Rechteinhabers einzuholen. Weitere Details zur Lizenz entnehmen Sie bitte der Lizenzinformation auf http://creativecommons.org/licenses/by/4.0/deed.de.
License URL: https://creativecommons.org/licenses/by/4.0/

issue-copyright-statement: © Springer-Verlag GmbH Deutschland, ein Teil von Springer Nature 2021

==============================
Chagas disease is a zoonosis caused by Trypanosoma cruzi and transmitted by triatomine bugs. Autochthonous to Latin America, Chagas disease has spread globally through travel and migration. New drugs are needed urgently, in particular drugs that cure the chronic stage. This is where high-content imaging makes a key contribution: assays with fluorescent parasites in cell culture allow to determine pharmacodynamic parameters and to better assess the antichagasic potential of new molecules.

■ Im Jahr 1909 beschrieb der brasilianische Arzt Carlos Chagas eine seltsame neue Krankheit: Im Blut eines zweijährigen Mädchens, das Fieber hatte und eine Hepatosplenomegalie aufwies, entdeckte er begeißelte Einzeller [1]. Es waren Trypanosomen der gleichen Art, wie er sie zwei Jahre zuvor aus blutsaugenden Wanzen der Familie der Triatominen isoliert und nach seinem Mentor, Oswaldo Cruz, benannt hatte. Heute wissen wir gut Bescheid über die Chagas-Krankheit, kennen die Genomsequenz ihres Erregers *Trypanosoma cruzi*, verstehen die Biologie der sie übertragenden Triatominen – und dennoch ist die globale Lage prekärer denn je.

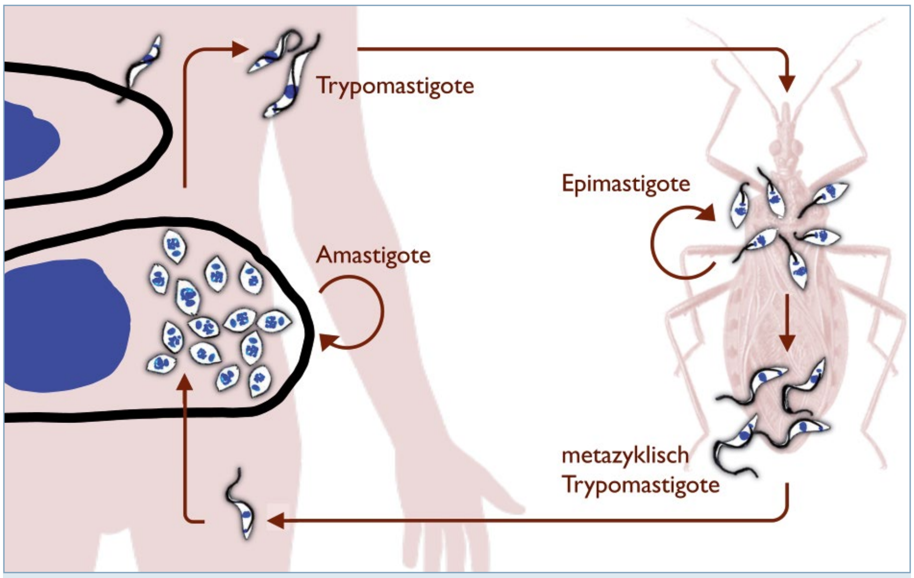

▲ **Abb. 1:** Der Lebenszyklus von *Trypanosoma cruzi* zwischen Säugetier und Raubwanze. Kreisförmige Pfeile kennzeichnen die proliferativen Stadien (intrazelluläre Amastigote im Säuger, extrazelluläre Epimastigote im Insekt). Die Trypomastigoten vermehren sich nicht, dafür sind sie an einen Wirtswechsel angepasst.

Von Zentral- und Südamerika aus, wo die Triatominen endemisch sind, wurde *T. cruzi* durch Migration und Reisetätigkeit weltweit verteilt. Auch in Abwesenheit des Vektors kann *T. cruzi* übertragen werden, vertikal von Mutter zu Kind oder horizontal über kontaminierte Blut- und Organspenden. Von den geschätzt sieben Millionen Infizierten weltweit, davon 100.000 in Europa, ahnen die meisten nicht, dass sie einen Parasiten beherbergen. Denn nach einer kurzen akuten Phase verläuft die Infektion über Jahrzehnte asymptomatisch. Nur etwa jeder dritte wird eine chronische Pathologie entwickeln, diese dann allerdings von lebensgefährlichem Ausmaß: Herzvergrößerung, Megaösophagus oder Megacolon bis zum Organversagen [2]. Die WHO schätzt die Zahl der jährlichen Todesfälle auf 10.000; die Dunkelziffer ist vermutlich groß.

Trotz verschiedener Initiativen, u. a. vom Helmholtz-Zentrum für Infektionsforschung in Braunschweig (www.cruzivax.eu), gibt es noch keine Impfung. Die Behandlung der Chagas-Krankheit beruht auf nur zwei, chemisch nah verwandten Arzneistoffen: Benznidazol und Nifurtimox. Beide haben zahlreiche Nebenwirkungen und sind während einer Schwangerschaft nicht zugelassen. Die Entwicklung besserer Medikamente ist somit von höchster Dringlichkeit, erlitt aber einen bitteren Rückschlag, nachdem die Spitzenreiter aus der Klasse der Azole im klinischen Versuch gescheitert waren. Diese Moleküle waren zwar höchst wirksam gegen *T. cruzi* in Zellkultur, vermochten aber nicht, die Chagas-Krankheit in der chronischen Phase zu heilen: 80 Prozent der behandelten Patienten erlitten Rückfälle [3]. Dieses ernüchternde Resultat zeigte auf, dass die Aussagekraft der *in vitro*-Tests verbesserungsbedürftig ist.

## *Trypanosoma cruzi* in der Raubwanze, im Säugetier und in Zellkultur

Der Lebenszyklus von *T. cruzi* umfasst blutsaugende Wanzen (Triatominae) als biologische Endwirte, Säugetiere als obligate Zwischenwirte und drei verschiedene Entwicklungsstadien des Parasiten: Trypomastigote, Amastigote und Epimastigote [1, 4]. Mit dem Blutmahl an einem infizierten Säugetier nimmt die Raubwanze Parasiten ein. Die meisten werden im Magen des Insekts verdaut, einige Trypomastigote überleben und differenzieren zu Epimastigoten. Diese wandern in den Darm, wo sie sich vermehren. Im Hinterdarm durchläuft der Parasit die Metacyclogenese, die Umwandlung von nichtinfektiösen Epimastigoten zu infektiösen Trypomastigoten, welche über den Kot ausgeschieden werden. Die Raubwanze defäkiert beim Blutsaugen und die Infektion des Warmblüters erfolgt durch Einreiben der Trypomastigoten in die Bisswunde oder durch deren Eindringen über die Schleimhäute. Die Trypomastigoten können alle Arten von kernhaltigen Zellen befallen. In der Wirtszelle werden sie zu unbegeißelten Amastigoten, vermehren sich und differenzieren schließlich wieder zu Trypomastigoten, welche nach Lyse der Wirtszelle entweder neue Zellen infizieren oder mit einer Blutmahlzeit von einer Raubwanze aufgenommen werden (**Abb. 1**).

Auf die unterschiedlichen Milieus (Insektendarm vs. Säugerzellen) muss auch in Zellkultur Rücksicht genommen werden. So werden die Epimastigoten unter axenischen Bedingungen bei 27 °C in serumhaltigem Leberinfusions-Tryptose(LIT)-Medium kultiviert. Amastigote werden bei 37 °C in Säugerzellen kultiviert, z. B. in *Microtus*-Embryonalfibroblasten in serumhaltigem RPMI-1640-Medium. Um den Zyklus aufrecht zu erhalten, werden die im Überstand frei schwimmenden Trypomastigoten wöchentlich geerntet und auf einen frischen Monolayer von Wirtszellen subpassagiert.

## Neue Möglichkeiten in der Wirkstofftestung dank High-Content-Mikroskopie

Die Entwicklung neuer Medikamente wird erschwert durch die Tatsache, dass die pathogene Form von *T. cruzi* intrazellulär ist [5]. In der Wirtszelle sind die Parasiten geschützt vor unserem Immunsystem und Medikamente müssen erst in das Gewebe und dann in die Wirtszelle eindringen, bevor sie wirken. Auch das Testen von Wirkstoffen im Labor wird erschwert, da intrazelluläre Parasiten nicht über die Verstoffwechselung von Resazurin in das fluoreszierende Resafurin quantifiziert werden können. Daher wurde Mitte der 1990er-Jahre ein transgener

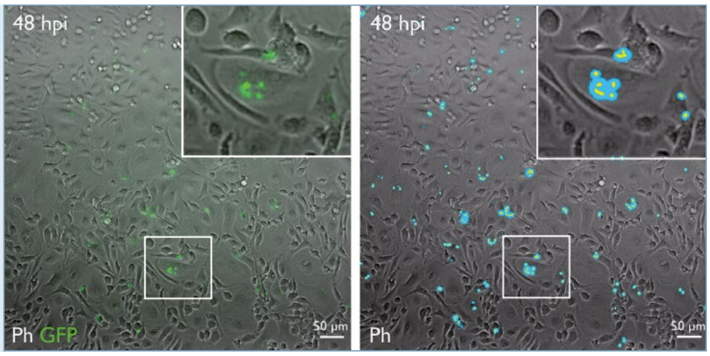

► **Abb. 2:** Grün fluoreszierende amastigote *Trypanosoma cruzi* in Makrophagen im *live imaging* (links) und Ergebnisdarstellung der automatisierten Auswertung (rechts). hpi: *hours post infection.*

*T. cruzi*-Stamm eingesetzt, der β-Galactosidase exprimierte [6]. Ein chromogenes Substrat sorgte für einen Farbumschlag, der mit der Anzahl Parasiten korrelierte, sodass diese nicht mehr in mühevoller Kleinarbeit nach Giemsa-Färbung mikroskopisch gezählt werden mussten. Somit ermöglichte der β-Galactosidase-Reporterstamm einen gesteigerten Durchsatz in der Wirkstofftestung. Doch dies konnte die aufwändige Auszählung von Amastigoten nicht vollständig ersetzen. Das Signal über ein ganzes Well gemessen lieferte keine Information bezüglich des Anteils infizierter Wirtszellen und vereinzelt überlebende Parasiten wurden nicht erfasst [7]. Gerade das erwies sich aber nach den ernüchternden Erfahrungen mit den Azolen als von höchster Wichtigkeit.

Als die High-Content-Mikroskopie leistungsfähiger und massentauglicher wurde, kam das auch der Medikamentensuche für die Chagas-Krankheit zugute. Denn nun konnten Parameter, wie die Zahl infizierter Wirtszellen und die Anzahl der Parasiten pro infizierter Wirtszelle, automatisch quantifiziert werden [8]. Messungen in Echtzeit wurden ermöglicht – sowie neue, komplexere Assayformate. Reversibilitätstests, bei denen die Wirksubstanz nach einiger Zeit ausgewaschen wurde, oder auch Kombinationstests von verschiedenen Wirkstoffen wurden durch die automatisierte Auswertung deutlich erleichtert.

## Replikationsrate und Kipppunkt als neue Parameter in der Wirkstofftestung

Für die Verwendung im *live imaging* haben wir eine *T. cruzi* Linie generiert [9], die das verstärkt grün fluoreszierenden Protein eGFP unter der Kontrolle eines ribosomalen Promoters und einer konstitutionell aktiven 3‘ untranslatierten Region [10] exprimiert. Dadurch werden die replizierenden Stadien fluoreszierend (**Abb. 2**). Dieser *T. cruzi*-Reporterstamm wurde in einem neuartigen Assaydesign eingesetzt, in dem pharmakodynamische Parameter bestimmt werden. Dazu wurde für jeden zu testenden Wirkstoff pro Replikat eine Platte mit nicht-replizierenden Wirtszellen ausgesät. Alle 24 Stunden wurde eine neue Reihe an Wells mit infektiösen Trypomastigoten des Reporterstamms infiziert. Nach jeweils 24 Stunden wurden die extrazellulären Trypomastigoten entfernt und ein Teil der Wells mit einer Verdünnungsreihe des Testwirkstoffs versetzt. So unterschieden sich benachbarte Reihen im Alter der Infektion und in der Dauer der Wirkstoffbehandlung um jeweils 24 Stunden. Am sechsten Tag wurde die Platte nach Infektion und Wirkstoffbehandlung versiegelt und in das High-Content-Mikroskop platziert. Dort wurden über 24 Stunden in jedem Well an neun Stellen alle vier Stunden fluoreszenz- und lichtmikroskopische Aufnahmen gemacht. Von diesen Aufnahmen wurde die Anzahl der Parasiten bestimmt. Im Anschluss wurden die Zellen fixiert. Mit Hoechst 33342 wurde die DNA von Wirtszellen und Parasiten angefärbt. So konnte überprüft werden, ob die Anzahl der grün fluoreszierenden Parasiten pro Bild mit der absoluten Anzahl an Parasiten übereinstimmte.

An jeder getesteten Stelle wurden während 24 Stunden sieben Messungen durchgeführt, jeweils mit vier Stunden Abstand. Die Entwicklung der Parasitenzahl wurde an jeder Stelle mithilfe eines exponentiellen Wachstumsmodells berechnet (**Abb. 3**, links). In den unbehandelten Kontrollen wurde ersichtlich, dass die Replikationsrate nach Infektion konsistent war, während der Parasitenstartwert variierte. In den behandelten Wells sank die Replikationsrate in Abhängigkeit von Wirkstoffkonzentration und Dauer der Behandlung. Der Graph der Änderungsrate über Wirkstoffkonzentration und Dauer der Behandlung zeigt für jeden Testwirkstoff ein individuelles Profil. Dabei lässt sich auf einen Blick ein Eindruck gewinnen, ob der Wirkstoff zu der Klasse der konzentrations-

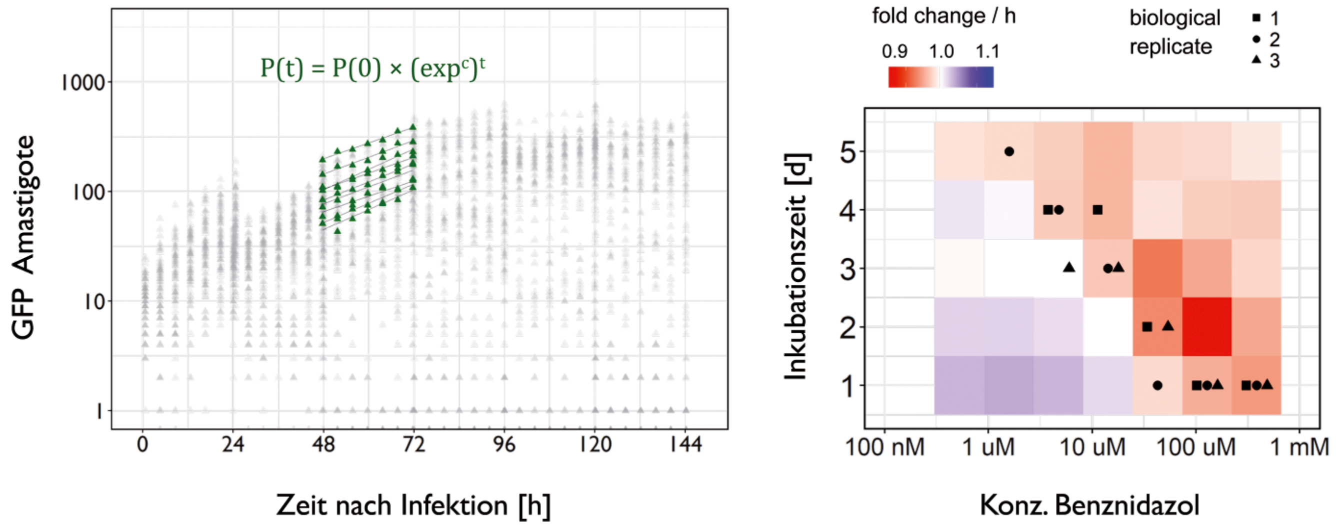

▲ **Abb. 3:** Über lokalisierte, exponentielle Wachstumskurven (links) wird die Änderungsrate der Parasitenzahl in Abhängigkeit von Konzentration und Inkubationszeit (rechts) der Testsubstanz bestimmt. Die Farbskala entspricht dem Wachstum der Parasiten pro Stunde (Replikationsrate). Der Kipppunkt (schwarze Symbole) zeigt an, wann die Replikationsrate von steigend (blau, >1) zu sinkend (rot, <1) gewechselt hat.

abhängig wirkenden oder der zeitabhängig wirkenden Substanzen gehört.

Darüber hinaus konnte so für jede getestete Konzentration eines Wirkstoffs der Zeitpunkt bestimmt werden, an dem die Replikationsrate signifikant unter 1 fiel und damit die Gesamtparasitenzahl abzunehmen begann (**Abb. 3**, rechts). Diesen Zeitpunkt nennen wir den Kipppunkt, da ab diesem Punkt die Replikation vom wirkstoffbedingten Parasitensterben dominiert wurde. Die Bestimmung der Änderungsrate und des daraus abgeleiteten Kipppunkts als neues Konzept in der Wirkstofftestung von einzelligen Parasiten wie *T. cruzi* wird bei der pharmakodynamischen Bewertung eines Medikamentenkandidaten helfen. Durch solche neuen High-Content-Formate können Wirkstoffe wie die Azole, die zwar aktiv gegen *T. cruzi* sind, aber eine ungünstige Pharmakodynamik aufweisen, frühzeitig eliminiert werden, bzw. vielversprechende Wirkstoffe oder Kombinationen davon frühzeitig erkannt werden. So leistet das High-Content-Imaging einen wertvollen Beitrag zur Wirkstoffentwicklung für die Chagas-Krankheit. ■


Funding note: Open Access funding provided by University of Basel.

Anna Fesser Jahrgang 1988. 2016–2019 Promotion über die Entwicklung neuer Wirkstofftests für die Chagas-Krankheit. Seit 2021 als Epidemiologin in der COVID-19-Sektion des Schweizer Bundesamts für Gesundheit.

Marcel Kaiser Jahrgang 1959. Seit 2000 Projektleiter und seit 2015 als Gruppenleiter in der Forschungseinheit Parasite Chemotherapy am Schweizerischen Tropen- und Public-Health-Institut; Schwerpunkt: Erforschung und Entwicklung neuer Wirkstoffe und Medikamente für die vernachlässigten tropischen Krankheiten, afrikanische Schlafkrankheit, Chagas-Krankheit, Leishmaniose und Malaria.

Pascal Mäser Jahrgang 1969. Seit 2012 Leiter der Forschungseinheit Parasite Chemotherapy am Schweizerischen Tropen- und Public-Health-Institut. Seit 2014 außerordentlicher Professor für Parasitologie und Protozoologie an der Universität Basel; Schwerpunkte: Wirkstoffe und Arzneimittel für Tropenkrankheiten, insbesondere die Schlafkrankheit, Chagas-Krankheit, Leishmaniose und Malaria.


Literatur

[1] Chagas C Nova tripanozomiaze humana: estudos sobre a morfolojia e o ciclo evolutivo do Schizotrypanum cruzi n. gen., n. sp., ajente etiolojico de nova entidade morbida do homem Mem Inst Oswaldo Cruz 1909 1 159 218 10.1590/S0074-02761909000200008
[2] Rassi A Jr. Rassi A Marin-Neto JA Chagas disease Lancet 2010 375 1388 1402 10.1016/S0140-6736(10)60061-X 20399979
[3] Molina I Gómez i Prat J Salvador F Randomized trial of posaconazole and benznidazole for chronic Chagas’ disease N Engl J Med 2014 370 1899 1908 10.1056/NEJMoa1313122 24827034
[4] Tyler KM Engman DM The life cycle of Trypanosoma cruzi revisited Int J Parasitol 2001 31 472 481 10.1016/S0020-7519(01)00153-9 11334932
[5] Francisco AF Jayawardhana S Lewis MD Biological factors that impinge on Chagas disease drug development Parasitology 2017 144 1871 1880 10.1017/S0031182017001469 28831944 PMC5729846
[6] Buckner FS Verlinde CL La Flamme AC Efficient technique for screening drugs for activity against Trypanosoma cruzi using parasites expressing beta-galactosidase Antimicrob Agents Chemother 1996 40 2592 2597 10.1128/AAC.40.11.2592 8913471 PMC163582
[7] Cal M Ioset JR Fügi MA Assessing anti-T. cruzi candidates in vitro for sterile cidality Int J Parasitol Drugs Drug Resist 2016 6 165 170 10.1016/j.ijpddr.2016.08.003 27639944 PMC5030316
[8] Engel JC Ang KKH Chen S Image-based high-throughput drug screening targeting the intracellular stage of Trypanosoma cruzi, the agent of Chagas’ disease Antimicrob Agents Chemother 2010 54 3326 3334 10.1128/AAC.01777-09 20547819 PMC2916317
[9] Fesser AF Braissant O Olmo F Non-invasive monitoring of drug action: A new live in vitro assay design for Chagas’ disease drug discovery PLoS Negl Trop Dis 2020 14 e0008487 10.1371/journal.pntd.0008487 32716934 PMC7419005
[10] Lewis MD Francisco AF Taylor MC A new experimental model for assessing drug efficacy against Trypanosoma cruzi infection based on highly sensitive in vivo imaging J Biomol Screen 2015 20 36 43 10.1177/1087057114552623 25296657 PMC4361455
